# Environmental and planetary health education for healthcare professionals: A social imperative

**DOI:** 10.1016/j.fhj.2025.100491

**Published:** 2025-12-03

**Authors:** Eleni Prifti, Vasiliki Andreou, Alexia Katsanidou

**Affiliations:** a Environmental Chemistry Department, National and Kapodistrian University of Athens, Greece; b European Lifestyle Medicine Organization, Switzerland; c Institute of Medical Education Research Rotterdam (iMERR), Netherlands; d GESIS-Leibniz Institute for Social Sciences, Germany; e Department of Sociology and Social Psychology, University of Cologne, Germany

**Keywords:** Health professional education, Planetary health, Medical curriculum, Professional competencies, Health equity, Sustainable healthcare

## Abstract

Recent literature highlights the urgent need for integrating environmental and planetary health into healthcare education, emphasising the impact of environmental exposures on disease and the interconnection between planetary and human health. However, these discussions often overlook critical social dimensions that make environmental and planetary health training for healthcare professionals not just beneficial, but essential. This paper argues that healthcare professionals possess significant social influence, can address gendered and socioeconomic health inequalities, play a key role in sustainable transitions, serve as critical barriers against pseudoscience and fear-based misinformation, improve crisis preparedness, address regional environmental health disparities, and contribute to lowering healthcare costs. We conclude with recommendations to integrate environmental and planetary health into medical education, continuous professional training and interdisciplinary collaborations.

## Introduction

The urgency of environmental crises, from climate change to pollution, has placed human health at the center of planetary health discourse.^1^^,^^2^ Recent articles have emphasised the importance of environmental and planetary health (EPH) training for healthcare professionals (HCPs), often highlighting the biomedical impacts of environmental exposures and calling for the inclusion of specific topics in health curricula.^3^, ^4^, ^5^ HCPs themselves identify the importance of EPH, but feel that they lack knowledge, tools and resources to address the issue.^6^ Despite these calls, EPH education remains largely absent from formal health professional training programmes. When present, it tends to be fragmented, limited in scope, or delivered without integration into clinical practice or local public health relevance.

Rather than identifying gaps in the content or structure of existing EPH programmes, this paper underscores how HCPs’ social influence and responsibilities necessitate EPH education as a core component of health training. It argues that EPH education must extend beyond clinical competencies and biomedical knowledge. It is a social imperative, driven by the unique societal roles that HCPs occupy as trusted communicators, public health advocates and crisis responders.

HCPs wield substantial authority in public discourse.^7^ Their ability to translate evidence into practice can influence lifestyle behaviours, meaning that their endorsement of environmental health principles can catalyse widespread behavioural shifts. Eurobarometer surveys (eg Special Eurobarometer 488, March 2019) consistently show that HCPs are among the most trusted sources of information, significantly ahead of political or administrative authority. Nevertheless, without proper EPH training, this authority remains underutilised – or, worse, misdirected – when HCPs unknowingly perpetuate outdated or incomplete perspectives on environmental determinants of health. Informed HCPs could serve as trusted messengers, guiding both individuals and policymakers towards evidence-based environmental health interventions.

To support this argument, the paper outlines six interconnected social dimensions that justify the urgent need for EPH training:

• Addressing the asymmetric impact of environmental health hazards across gender, age, and socioeconomic groups by advocating for equity-informed health interventions.
• Combating pseudoscience and fearmongering by serving as sources of evidence-based information and fostering public literacy.
• Responding to regional environmental health inequalities by recognising how environmental burdens are distributed.
• Strengthening HCPs’ role in advocacy for influencing institutional policies and promoting public engagement in sustainability transitions.
• Promoting adaptive expertise for crisis response.
• Reducing healthcare costs by shifting towards a prevention-focused model.

### The asymmetric impact of environmental hazards

Environmental exposure does not affect populations equally. Women and children are disproportionately vulnerable to environmental risks such as toxic exposures, heat stress and endocrine-disrupting chemicals.^8^ Indicatively, pregnant women and young children are biologically more susceptible to the harmful effects of air pollution,^9^ while socioeconomically disadvantaged populations often face compounded risks by living in closer proximity to highly polluted environments.^10^

Despite the evidence on disparities in health outcomes, the role of EPH in addressing these is often overlooked. EPH training should enable HCPs to appropriately recognise and address these vulnerabilities, contributing to more equitable healthcare interventions, outcomes and policies. This would lead to tailored medical advice and advocacy for policies that mitigate the specific environmental risks faced by vulnerable populations.

### Combating pseudoscience and fearmongering

The absence of EPH training among HCPs combined with the abundance of misinformation in social media fuels the spread of pseudoscientific narratives regarding toxic exposures, with radiophobia being a characteristic example.^11^ Without the ability to critically assess environmental risks, many HCPs inadvertently amplify misinformation, unnecessarily alarming patients about low-risk exposures while neglecting truly hazardous ones.

This has led to the proliferation of pseudoscientific ‘detoxification’ practices, fear-based dietary restrictions and misleading health claims.^12^ Systems level and critical thinking competencies within EPH education would equip HCPs with the tools to distinguish real risks from exaggerated or false narratives, fostering a rational, evidence-based approach to environmental health communication.^13^

### Addressing regional health inequalities

While environmental and planetary health challenges affect everyone, their impact is unevenly distributed across regions. In countries of the Global South with severe pollution levels, like India, where air pollution is a major contributor to respiratory and cardiovascular diseases, EPH education for HCPs is even more crucial.^14^ Unfortunately, at the moment, the vast majority of EPH programmes are designed from institutions of the Global North and exclusively in English, limiting accessibility and contextual relevance.^3^

HCPs in high-pollution areas should be equipped to:

• Recognise early symptoms of pollution-related illnesses (eg lung disease, neurodevelopmental delays).
• Guide patients on evidence-based protective measures (eg air filtration, exposure reduction).
• Advocate for policy changes by providing data on pollution-related hospitalisations and mortality.
• Understand and monitor critical environmental infrastructure in their communities, such as drinking water quality, waste management, and proximity to industrial pollution sources, enabling them to connect environmental exposures with clinical symptoms more accurately.

By integrating EPH education in these regions, HCPs can respond effectively to region-specific environmental health threats, ensuring that healthcare interventions are not only evidence-based but also contextually grounded in the lived realities of the populations they serve.

### Promoting sustainability transitions

A visible consequence of inadequate EPH education is HCPs’ resistance to sustainability-driven healthcare changes. This is particularly evident in the adoption of plant-based diets, which are strongly supported by scientific consensus for both human and planetary health.^15^^,^^16^ However, the lack of EPH education and targeted training in plant-based nutrition leads many HCPs to discourage its adoption, often due to outdated beliefs about the adequacy of plant-based proteins and concerns over the bioavailability of essential micronutrients such as iron and calcium.^17^

This resistance is further compounded by low participation in lifelong learning programmes and an overreliance on unverified sources, such as social media. Recent evidence shows that a significant number of HCPs rely on social media for clinical information and are vulnerable to misinformation – especially when lacking structured continuing education.^18^ Without robust EPH training and continuing professional development, HCPs risk becoming barriers rather than facilitators of sustainable dietary transitions, perpetuating misinformation and hindering public adoption of healthier, more sustainable lifestyles.

Proper training in nutrition and environmental sustainability would enable HPs to provide evidence-based recommendations, counter misinformation, and reinforce trust in science-based dietary guidance.

### Strengthening public health preparedness and crisis response

Environmental crises due to climate change, industrial pollution, or emerging infectious diseases linked to ecological disruption are becoming major public health emergencies. HCPs need EPH training to anticipate, prevent, and manage these crises effectively.^19^ Various environmental crises are already challenging HCPs’ practices. Climate-related disasters (heatwaves, floods, wildfires) are becoming more common, yet HCPs often lack preparedness training.^20^ Vector-borne diseases (dengue, malaria) are shifting due to climate change, requiring HCPs to recognise new environmental health patterns.^21^ Industrial accidents and chemical spills require HCPs to communicate risks responsibly while preventing public panic.^22^

To ensure a more effective and equitable public health response, EPH-trained HCPs should be actively included in municipal, regional and state-level planning and preparedness committees. Their medical expertise, combined with an understanding of environmental determinants of health, positions them to contribute meaningfully to disaster planning, risk communication strategies, and community resilience building. This integration would help ensure that environmental health concerns are prioritised in broader public health and infrastructure policies.

### Reducing healthcare costs through prevention

Environmental factors are major contributors to preventable diseases. Yet without proper training in EPH, HCPs often lack the competencies needed to identify, link, address and clearly communicate these risks. Consequently, care remains reactive, focusing on treating illness after it occurs, rather than preventive, contributing to rising disease burdens and healthcare costs.^23^

For instance, air pollution-related diseases cost European healthcare systems billions annually due to increased hospital admissions and lost productivity.^24^ EPH education equips HCPs with essential competencies to recognise environmental risk factors and guide patients in reducing their exposures, whether by advising on how to avoid peak pollution times, choose low-emission transport options, or select safer personal care and dietary products. This kind of informed, prevention-oriented care not only protects individual health but also reduces long-term system-wide costs.

## Recommendations

To address the urgent need for EPH integration in healthcare, we propose a set of concrete actions aligned with global competency-based education frameworks and the realities of adult learning. The CanMEDS model, one of the most widely use competency-based frameworks in health professions education, has already begun recognising planetary health as a core competency, highlighting the need for doctors to understand and act upon the interconnectedness of human and environmental systems.^25^ Moreover, professionals may struggle to apply scientific knowledge to real-life decision-making if their training does not bridge theory and lived experience.^26^ EPH education must therefore be practical, interdisciplinary and lifelong. Our recommendations aim to empower health professionals with the tools and context-specific knowledge necessary to address environmental determinants of health, both in clinical and public health practice.

We recommend:

1. Conducting targeted research on the current state of EPH literacy among health professionals, including their ability to apply environmental knowledge in real-world healthcare settings.
2. Engaging diverse stakeholder groups (ie HCPs, public health experts, community organisations and patients) in co-creating educational strategies to ensure EPH education reflects the realities of local communities, and the needs of the broader health system.
3. Integrating EPH into continuing professional development programmes to support lifelong learning and ensure that existing HCPs remain current with emerging environmental and planetary health threats.
4. Embedding EPH into medical and health curricula from the undergraduate level, framing it not as an elective topic but as a fundamental competency in line with competency-based medical educations such as CanMEDS.
5. Fostering interdisciplinary collaboration across environmental sciences, healthcare and social sciences to build comprehensive training that reflects the complexity of real-life exposures and inequalities.
6. Developing accessible, technology-driven, evidence-based clinical tools that support HCPs in assessing environmental health risks and delivering practical, fear-free guidance to patients; tools that can counter pseudoscience and improve public health messaging.
7. Strengthening government policy and investment to embed EPH within HPEs, aligning national strategies and funding to enhance preparedness and resilience.

## Conclusion

As environmental and planetary health challenges grow in scale and urgency, the role of HCPs must evolve accordingly. This paper argues that EPH education is not only medically relevant, but socially imperative. HCPs are uniquely positioned as trusted messengers, advocates and frontline responders whose actions extend far beyond the clinic. Without adequate training in EPH, their potential to influence behaviour, shape public understanding and advocate for systemic change remains untapped, or worse, misdirected.

By embedding EPH education into medical curricula and professional development programmes, we can equip HCPs to respond more effectively to the social and environmental determinants of health. This includes, for instance, a GP recognising patterns of asthma exacerbations during local pollution events and advocating for municipal air quality interventions, or a midwife supporting pregnant women living near industrial zones by integrating environmental risk awareness into prenatal care. These actions demonstrate how EPH-literate professionals can address health disparities and drive locally relevant, evidence-based changes.

If we are to build resilient, equitable and sustainable health systems, we must reframe EPH not as an optional add-on, but as a foundational component of healthcare professionalism in the 21st century. Only then can HCPs fully serve their dual role: protectors of individual health and stewards of planetary wellbeing as the natural human habitat.

## CRediT authorship contribution statement

**Eleni Prifti:** Writing – original draft, Resources, Conceptualization, Resources. **Vasiliki Andreou:** Writing – review & editing, Validation. **Alexia Katsanidou:** Writing – review & editing, Supervision.

## Declaration of competing interest

The authors declare that they have no known competing financial interests or personal relationships that could have appeared to influence the work reported in this paper.
